# Development of the Fabry Disease Patient-Reported Outcome (FD-PRO): a new instrument to measure the symptoms and impacts of Fabry Disease

**DOI:** 10.1186/s13023-021-01894-2

**Published:** 2021-06-25

**Authors:** Alaa Hamed, Pronabesh DasMahapatra, Nicole Lyn, Chad Gwaltney, Robert J. Hopkin

**Affiliations:** 1 Sanofi Genzyme, 50 Binney Street, Cambridge, MA 02142 USA; 2 Gwaltney Consulting, Westerly, RI USA; 3 Cincinnati Children’s Hospital Medical Center and University of Cincinnati College of Medicine Department of Pediatrics, Cincinnati, OH USA

**Keywords:** Fabry disease, Patient-reported outcome, FD-PRO, Clinical outcome assessment, Rare disease, Patient experience, Qualitative interviews

## Abstract

**Background:**

The systematic collection of disease-specific symptoms and impacts on the lives of patients with Fabry Disease (FD) can offer unique insights into the patient experience, yet no disease-specific tool to measure FD symptoms exists. This study describes the development of the Fabry Disease Patient-Reported Outcome (FD-PRO).

**Methods:**

A targeted literature search, interviews with key opinion leaders (KOLs), and concept elicitation (CE) interviews with patients identified the most frequent signs and symptoms associated with FD and their impact on daily life. Cognitive interviews evaluated patients’ ability to understand the FD-PRO instructions and respond to the items on the draft FD-PRO instrument.

**Results:**

The targeted literature search identified key signs and symptoms in domains that were confirmed in KOL interviews. In CE interviews with 37 treated and treatment-naïve patients, neuropathic pain symptoms (95% treated, 82% treatment-naïve), temperature intolerance (95% treated, 88% treatment-naïve), energy difficulties (95% treated, 94% treatment-naïve), hearing/vision impairment (95% treated, 71% treatment-naïve), and gastrointestinal symptoms (80% treated, 59% treatment-naïve) were most frequently mentioned. Results were similar for men and women in both treated and treatment-naïve groups. While treatment-naïve patients in general expressed fewer and milder symptoms compared to treated patients, the overall sets of symptoms expressed by the two groups were similar. The most severe symptoms were neuropathic pain, stomach pain, burning pain, and fatigue. The most bothersome symptoms were stomach pain, breathing difficulty, fatigue, neuropathic pain, and constipation. The most frequent impacts were in the work/school limitations domain for both treated and treatment-naïve patients. The impacts with the highest difficulty ratings were stress, limited outdoor activity, and guilt. Cognitive interviews with 14 treated and treatment-naïve patients resulted in the refinement of FD-PRO items and language.

**Conclusions:**

The FD-PRO is a novel, disease-specific instrument that measures the patient experience in Fabry disease. Such tools are valuable in capturing the burden of disease in patients with FD and demonstrating the value of treatment in clinical trials.

## Background

Fabry Disease (FD) is a rare genetic disorder caused by mutations in the lysosomal enzyme α-galactosidase A (α-Gal A) that lead to the progressive accumulation of globotriaosylceramide (GL-3) and other products in the lysosomes of cells [1, 2]. Over time, the accumulation of these products damages cells and leads to progressive and irreversible organ damage, typically involving the nervous system, endothelium, kidney, and heart, as well as decreased life expectancy [1, 2]. However, before those events occur, patients with FD experience a wide range of debilitating symptoms including neuropathic pain, gastrointestinal (GI) discomfort, a decreased ability to sweat, heat intolerance, angiokeratoma, and fatigue that negatively impact their lives [1].

The systematic collection of disease-specific symptoms and the impact of these symptoms on the lives of patients with FD can offer unique insights into the patient experience. Since symptoms and disease impacts are feelings that are experienced by the patient, not observable by clinicians, and not optimally evaluated in a standard clinical trial setting, they are best measured by asking the patient to evaluate them using disease-specific patient-reported outcome (PRO) instruments [3]. PRO instruments can be used to evaluate the patient experience in observational studies and during the daily lives of patients. Such instruments can also help with measuring the benefit of new therapies from the patient point of view (i.e., symptom alleviation or improvement).

Improving and preventing symptom burden is an important clinical goal and treatments for FD can alleviate symptoms and reduce the impact that they have on health-related quality of life (HRQOL) [2]. A systematic literature review of available studies examining HRQOL measurement identified the need for a FD-specific questionnaire to accurately assess HRQOL in patients with FD [4]. With a PRO questionnaire that assesses symptom alleviation, clinical trials evaluating treatments for FD may be able to demonstrate improvement in symptom-related outcomes in a shorter timeframe compared to improvements in outcomes measuring morbidity and mortality. Therefore, symptomatic improvement in a clinical trial may indicate treatment efficacy and clinical benefit for a therapeutic product.

Though other PROs have been developed in FD, these questionnaires are specific in scope or population (e.g., treatment expectations, GI symptoms, children with FD) [5–7]. Therefore, there is a need to implement a PRO that assesses the broader spectrum of symptoms of FD. As no disease-specific PRO has been developed in FD that assesses the broad spectrum of severity of symptoms, there is a gap in the ability to provide a structured measurement of symptoms of FD. The objective of this study was to understand the perspective of patients with FD and develop a FD-specific PRO.

## Methods

The content validity of the instrument was established based on the process outlined by the Food and Drug Administration (FDA) Guidance for Industry and expert guidelines [3, 8]. The Fabry Disease Patient-Reported Outcome (FD-PRO) instrument was developed using qualitative research methods which included a targeted literature review, interviews with key opinion leaders (KOLs), and concept elicitation (CE) interviews with patients to identify the symptoms most relevant to patients with FD and the impact that these symptoms have on their lives. The qualitative research was followed by cognitive debriefing interviews that assessed the ability of patients to understand and respond to the PRO instrument. The qualitative research and cognitive interviews informed the refinement of the FD-PRO and the psychometric properties have been established in an observational study [9].

### Concept elicitation

CE was comprised of 3 main steps: a targeted literature review, interviews with KOLs, and patient interviews. The goal of the targeted literature review was to identify the most relevant and most important signs, symptoms, and impacts of FD from the patient perspective. The identified sign, symptom, and impact concepts were used to develop the FD-PRO instrument. The targeted literature review was conducted in PubMed and ClinicalTrials.gov in May and June 2013 using the key word “Fabry” and additional search terms (e.g., sign, symptom, quality of life). The targeted literature review was used to create the interview guides prior to patient and KOL interviews.

The interviews with 7 KOLs were conducted to identify the priority signs, symptoms, and impacts of FD that were frequently experienced by patients, used as endpoints in clinical trials, and/or improved by enzyme replacement therapy (ERT). Telephone interviews were conducted with KOLs from the United States (US) (n = 3), Brazil (n = 1), the United Kingdom (UK) (n = 1), and Denmark (n = 1). In addition, an in-person interview was conducted with a KOL from Japan (n = 1). The interviews evaluated the experiences of patients dealing with the symptoms of FD, how symptoms change over time, and how the experience differed by age, sex, and treatment.

Thirty-seven CE interviews with patients from the US aimed to qualitatively assess the experiences that patients had with the signs and symptoms of FD and the impact that these signs and symptoms had on their daily lives. The CE interviews included patients ≥ 18 years of age with a diagnosis of Fabry Disease. Male patients’ diagnoses were confirmed by leukocyte α-GAL A activity assay of < 4 nmol/h/mg OR documented plasma α-GAL A activity < 1.5 nmol/h/mL AND a documented causative α-GAL A mutation. Female patients’ diagnoses were confirmed by a documented causative α-GAL A mutation. Both treated and treatment-naïve as well as classic and late-onset patients were included in the study. To be eligible for enrollment as a treated patient, treatment with Fabrazyme 1 mg/kg q2w for ≥ 6 months was required.

The study excluded patients who had a history of, or active, clinically significant organ disease (except for symptoms related to FD) that, in the opinion of the Investigator, precluded participation in the study or interfered with the interpretation of the interview data. Patients were also excluded if they had a positive test result for hepatitis B surface antigen (HBsAg), anti-hepatitis C virus (anti-HCV) antibodies, and/or anti-human immunodeficiency virus 1 and 2 antibodies (anti-HIV1 and anti HIV2 antibodies), or had participated in a study of an investigational drug within 30 days of the start of the study.

A semi-structured CE interview guide was used to identify the terms that patients used to describe the signs, symptoms, and impacts associated with their condition. Interviews were transcribed and coded in ATLAS.ti (version 7.0) to identify themes in the data [10]. The significance of FD-related symptom and impact concepts was assessed by the frequency of patients’ mention of each concept. Saturation of FD symptom and impact concepts was achieved by first creating 4 groups of transcripts, organized chronologically. The codes that were derived from each group were compared to those in the previous group until no new concepts were identified within a group of transcripts. A conceptual model was developed using the concepts reported during interviews to highlight the most frequently reported symptoms and impacts of FD.

### Cognitive debriefing

Cognitive debriefing interviews were conducted to evaluate the ability of patients with FD to understand the FD-PRO instructions and respond to the items on the draft FD-PRO instrument. These interviews also helped to identify potentially problematic terminology that prevented patients from understanding the instructions and responding to the items of the instrument.

Inclusion and exclusion criteria for the cognitive interviews were the same as for the CE interviews. Cognitive interviews were conducted in 3 waves, with revisions at each wave, to produce the final list of concepts for the FD-PRO. Overall, 14 patients participated in the cognitive debriefing interviews and all patients were from the US.

## Results

### Targeted literature search

The targeted searches identified 677 abstracts, from which 30 articles were identified for full text review. Review of studies on the ClinicalTrials.gov website identified 84 studies, of which 16 had FD signs, symptoms, or impacts as primary or secondary outcome measures. The signs/symptoms and impacts relevant to patients with Fabry Disease identified by these searches are listed in Table 1.

**Table 1:** Concepts identified in targeted literature review

Signs/symptoms	Impacts
Neuropathic pain (acroparesthesia, Fabry crisis pain, and chronic continuous pain in hands and feet)	Physical activities, including exercise and sports
Gastrointestinal symptoms (abdominal pain, diarrhea, constipation, nausea, and vomiting)	Work and school productivity
	Impaired relationships (social, intimate, and family)
Impaired sweating (hypohidrosis and anhidrosis)	
	Impaired emotional functioning, particularly depression and anxiety
Angiokeratoma	
Hearing impairment (hearing loss, tinnitus)	
	General quality of life
Heat intolerance	
Lymphedema	
Headache	
Renal complications (proteinuria, need for dialysis and/or transplant)	
Cardiac complications (chest pain, palpitations, CHF, and MI)	
Cerebrovascular complications (stroke and TIA)	
Ophthalmological issues (corneal verticillate and tortuous vessels)	
Fatigue, tiredness, or excessive sleepiness	

### KOL interviews

The KOLs confirmed that while symptom experience varies among patients, all the symptoms identified in the targeted search were relevant, and that the same PRO instrument could be used to capture the experience of both men and women and of both treated and treatment-naive patients. The KOLs noted that renal, cardiac, and cerebrovascular complications are not commonly experienced in early disease, and that symptoms such as angiokeratomas and corneal verticillata are common, but less bothersome than other symptoms. Impacts including productivity, work or school performance, activity limitations, and perception of health were mentioned by KOLs and further explored in the CE interviews.

### CE interviews

A total of 37 patients with FD met the eligibility criteria and were interviewed at 6 US clinical sites. Patients ranged from 23 to 74 years of age with a mean age of 45.7 years; 22 (59.5%) patients were women, and 32 (86.5%) were White. Thirty-four patients had classic FD and 3 patients had late-onset FD (Table 2).

**Table 2:** Baseline demographic and clinical characteristics of concept elicitation and cognitive interview participants

Characteristic	Concept elicitation participants			Cognitive interview participants
	Treated group (n = 20)	Treatment-naïve group (n = 17)	All (N = 37)	All (N = 14)
Age (years), mean ± SD	45.7 ± 12.1	43.7 ± 13.8	45.7 ± 12.9	48.9 ± 15.8
Female, n (%)	11 (55.0)	11 (64.7)	22 (59.5)	8 (53)
*Phenotype*				
Classic, n (%)	19 (95.0)	15 (88.2)	34 (91.9)	N/A
Late-onset, n (%)	1 (5.0)	2 (11.8)	3 (0.1)	N/A
*Marital status, n (%)*				
Married/living as married	14 (70.0)	11 (64.7)	25 (67.6)	5 (36)
Widowed	0	1 (5.9)	1 (2.7)	2 (14)
Separated/divorced	2 (10.0)	1 (5.9)	3 (8.1)	4 (29)
Never married	4 (20.0)	4 (23.5)	8 (21.6)	3 (21)
Post-secondary education, n (%)	13 (65.0)	13 (76.5)	26 (70.2)	12 (86)
*Employment status, n (%)*				
Employed full-time, part-time, self-employed	13 (65.0)	12 (70.6)	25 (67.6)	7 (50)
Out of work	1 (5.0)	2 (11.8)	3 (8.1)	2 (14)
Homemaker	1 (5.0)	1 (5.9)	2 (5.4)	2 (14)
Retired	1 (5.0)	2 (11.8)	3 (8.1)	1 (7)
Unable to work	4 (20.0)	0	4 (10.8)	2 (14)
*Race, n (%)*				
White	15 (88.2)	17 (85.0)	32 (86.5)	14 (100)
Black	2 (11.8)	2 (10.0)	4 (10.8)	0
Other	0	1 (5.0)	1 (2.7)	0
Hispanic, Latino, or Spanish origin, n (%)	2 (11.8)	1 (5.0)	3 (8.1)	0
Time since diagnosis, years, mean ± SD	14.5 ± 13.7	3.6 ± 7.6	9.6 ± 12.3	12.0 ± 9.6
Time since confirming genetic test, years, mean ± SD	11.3 ± 13.7	2.5 ± 3.4	7.4 ± 10.9	N/A
Treated with ERT ≥ 3 years, n (%)	12 (60.0)	0	12 (32.4)	N/A
Ever received treatment (Replagal, SRT, or Fabrazyme	N/A	N/A	N/A	12 (86)
Treated with Fabrazyme ≥ 6 months, n (%)	N/A	N/A	N/A	11 (79)

*CE* concept elicitation, *ERT* enzyme replacement therapy, *N/A* not applicable, *SD* standard deviation

As noted in “Methods”, the interview transcripts were ordered by date into 4 groups for determination of saturation. Each group was assessed versus the previous group for the appearance of new concepts. Concept saturation was achieved as no new concepts were identified in the last group of the patient sample. A total of 96.4% of concepts were identified in the first group, 3.2% were identified in the second group, and 2.2% were identified in the third group; no new concepts were identified in the fourth group.

#### Signs and symptoms of FD

Neuropathic symptoms, temperature intolerance, energy difficulties, digestive/GI symptoms, and hearing/vision impairment were most frequently mentioned by patients (Table 3). Results were similar between male and female treated patients, and between male and female treatment-naïve patients. While treatment-naïve patients in general expressed fewer and milder symptoms compared to treated patients, the overall sets of symptoms expressed by the two groups were similar. All concepts expressed by treatment-naïve patients were also expressed by treated patients with the exception of coughing and excessive sweating. Eight symptom concepts were expressed only by treated patients and not by treatment-naïve patients. Even so, these symptoms were rare in the treated group: localized fever, temperature insensitivity, physical weakness, earache, dry eyes, rash, other skin problems (unspecified), and speaking difficulties.

**Table 3:** Symptom concept frequencies, severity, and most bothersome ratings

Symptom concepts	Treated group (n = 20)			Treatment-naïve group (n = 17)		
	Frequency, %	Severity rating, mean (n)	Bothersome rating, mean (n)	Frequency, %	Severity rating, mean (n)	Bothersome rating, mean (n)
*Neuropathic pain symptoms*	**95.0**			**82.4**		
Burning pain	**80.0**	**7.9 (9)**	*5.0 (7)*	*47.1*	**7.3 (6)**	*6.6 (5)*
Crisis pain	*60.0*	**8.7 (3)**	*5.8 (5)*	*29.4*	**7.5 (2)**	**7.5 (2)**
Neuropathic pain	10.0	**10.0 (1)**	*6.0 (1)*	0.0	–	–
Numbness	*70.0*	*5.8 (1)*	*5.8 (5)*	23.5	**7.3 (3)**	**8.0 (3)**
General pain	**75.0**	**8.0 (7)**	*5.8 (4)*	41.2	**9.5 (2)**	**10.0 (2)**
Tingling	45.0	*5.3 (4)*	3.0 (3)	**52.9**	*6.3 (7)*	*5.3 (7)*
*Other pain symptoms*	**90.0**			**64.7**		
Headache/migraine	**55.0**	*6.8 (6)*	*5.7 (6)*	52.9	*5.3 (6)*	*5.0 (6)*
Localized pain	40.0	*8.3 (3)*	*6.5 (2)*	23.5	*6.8 (5)*	**7.0 (4)**
Whole body pain	45.0	*5.9 (7)*	**7.3 (4)**	35.3	**9.5 (2)**	**9.0 (2)**
*Digestive/gastrointestinal symptoms*	**80.0**			**58.8**		
Constipation	15.0	*6.5 (2)*	**8.7 (3)**	23.5	**7.0 (3)**	**7.0 (3)**
Cramping	20.0	**7.8 (4)**	*5.8 (4)*	11.8	**8.0 (1)**	–
Diarrhea	**75.0**	*6.8 (12)*	*5.2 (9)*	35.3	*6.8 (6)*	*6.5 (4)*
Digestion and heartburn	25.0	–	–	11.8	–	–
Gas and bloating	15.0	**10.0 (1)**	**10.0 (1)**	23.5	**7.0 (1)**	*6.0 (1)*
Nausea	15.0	–	–	11.8	**8.0 (1)**	**8.0 (1)**
Stomach pain	*30.0*	**10.0 (1)**	**10.0 (1)**	35.3	**7.5 (4)**	**9.8 (4)**
Upset stomach	*35.0*	*6.5 (2)*	*5.3 (3)*	35.3	–	–
Urgency	*35.0*	**8.3 (6)**	*6.8 (4)*	11.8	**7.0 (1)**	2.5 (2)
Vomiting	10.0	–	*5.0 (1)*	5.9	2.0 (1)	**10.0 (1)**
*Temperature intolerance and change*	**95.0**			**88.2**		
Cold intolerance	*55.0*	*5.6 (7)*	*4.4 (5)*	*58.8*	**7.9 (7)**	*6.1 (8)*
General fever	*45.0*	*6.5 (2)*	4.7 (3)	11.8	–	–
Excessive sweating	0	**8.0 (1)**	**10.0 (1)**	17.6	5.6 (5)	5.0 (2)
Heat intolerance	**85.0**	**7.4 (14)**	*6.7 (14)*	47.1	**7.4 (10)**	*6.8 (5)*
Lack of sweating	*65.0*	*6.6 (11)*	3.2 (11)	52.9	*5.2 (5)*	2.2 (9)
Localized fever	5.0	**10.0 (1)**	**7.0 (1)**	0.0	–	–
Temperature insensitivity	5.0	**10.0 (1)**	2.0 (1)	0.0	–	**7.0 (1)**
*Edema*	**55.0**			41.2		
Swelling	**55.0**	6.4 (10)	6.4 (11)	41.2	4.8 (5)	4.2 (5)
Other edema	5.0	–	**10.0 (1)**	0.0	–	–
*Energy difficulties*	**95.0**			**94.1**		
Fatigue	*70.0*	**7.7 (9)**	**9.0 (9)**	47.1	*6.8 (4)*	**7.4 (5)**
Low stamina	15.0	*6.0 (1)*	*6.0 (1)*	11.8	–	–
Low or no energy	*40.0*	*6.0 (1)*	*5.5 (2)*	41.2	*6.0 (1)*	**8.0 (2)**
Physical weakness	*30.0*	–	–	0.0	–	–
Tiredness	**90.0**	**6.9 (11)**	**7.2 (10)**	**76.5**	**7.4 (10)**	**7.5 (11)**
*Hearing and vision problems*	**95.0**			*70.6*		
Earache	5.0	*6.0 (2)*	**9.5 (2)**	0.0	–	–
Hearing loss	**75.0**	**7.1 (8)**	*6.3 (8)*	17.6	*4.0 (1)*	–
Tinnitus	*50.0*	*3.8 (9)*	*3.9 (9)*	*47.1*	*5.8 (8)*	**7.5 (11)**
Dry eyes	10.0	**8.0 (1)**	**10.0 (1)**	0.0	–	–
Sight impairment	*50.0*	*6.6 (5)*	*6.2 (5)*	17.6	*5.5 (2)*	*5.3 (3)*
Corneal whorling	*30.0*	0.0 (1)	–	11.8	–	0.0 (1)
Other eye problems	5.0	–	–	11.8	–	–
*Skin problems*	**80.0**			23.5		
Angiokeratomas	20.0	2.0 (1)	2.0 (1)	23.5	–	–
Rash	*45.0*	*4.9 (10)*	2.3 (11)	0.0	3.3 (3)	2.0 (3)
Other skin problems	*30.0*	–	–	0.0	*6.0 (1)*	*5.0 (1)*
*Cardiac problems*	*70.0*			**52.9**		
General heart problems	20.0	–	1.0 (1)	*41.2*	*5.0 (2)*	*4.5 (4)*
Heart damage	*35.0*	*5.0 (5)*	**7.2 (6)**	11.8	**7.5 (2)**	*4.0 (4)*
Heart palpitations/arrhythmia	*45.0*	*6.3 (5)*	*6.0 (6)*	5.9	*4.8 (4)*	2.5 (4)
*Respiratory problems*	*50.0*	**–**	**–**	**64.7**		
Breathing difficulty	*35.0*	**9.0 (2)**	**9.7 (3)**	41.2	*5.0 (1)*	**8.0 (1)**
Coughing	25.0	**9.0 (1)**	**7.0 (1)**	41.2	*6.0 (1)*	*5.0 (1)*
Other respiratory problems	**95.0**	**9.0 (1)**	**9.0 (1)**	17.6	–	–
*Additional symptoms*	15.0			*88.2*		
Blood pressure	15.0	**8.0 (1)**	*5.0 (1)*	5.9	–	–
Cerebrovascular symptoms	20.0	–	–	5.9	–	–
Patient reports of cerebrovascular events	*30.0*	**8.3 (3)**	**7.3 (3)**	5.9	**10.0 (1)**	**10.0 (1)**
Dizziness or lightheadedness	*50.0*	**8.0 (3)**	*5.7 (3)*	*29.4*	**8.3 (3)**	**8.7 (3)**
Feeling ill	*40.0*	–	–	17.6	–	–
Patient-expressed kidney signs and symptoms	*30.0*	3.3 (4)	*5.4 (7)*	*41.2*	**8.0 (1)**	*4.1 (8)*
Patient reports of kidney-related clinical findings	*40.0*	–	–	*47.1*	–	–
Musculoskeletal problems	*40.0*	*6.3 (3)*	*5.3 (3)*	11.8	**10.0 (1)**	**7.0 (2)**
Speaking difficulties	20.0	–	–	0.0	–	–
Urinary problems	*35.0*	2.0 (2)	*5.5(2)*	11.8	**9.3 (3)**	*6.3 (3)*
Other symptoms	25.0	–	–	5.9	–	–

Italics indicate higher expressions. Bold indicates highest expressions

Bothersome and severity ratings range from 0 = not severe/bothersome at all, to 10 = extremely severe/bothersome

After patients spontaneously described their symptoms during the CE interviews, interviewers probed about symptoms not mentioned using the interview guide. Concepts that were most frequently expressed spontaneously were tiredness, fatigue, burning pain, heat intolerance, and lack of sweating; these can be considered to carry more relevance than symptoms expressed after probing.

##### Neuropathic pain

The most frequently reported concept overall was burning pain which was reported by 80% of treated patients and 47.1% of treatment-naïve patients. A patient described his/her burning pain as “a really hot sensation and in my hands all the way to the fingertips to the palms and really it is just intense heat, intense pain” (Participant 8106). Burning pain was worsened by triggers, such as exercise or extreme temperatures. One patient experienced burning pain in the hands during cold weather, stating “they are burning, like they are on fire … It's just like I touched a hot, that cold ice stuff [dry ice]” (Participant 8104). While burning pain was similar in presentation to neuropathic pain, patients primarily characterized their pain as “burning” which resulted in the separate concept. The term “neuropathic pain” was mentioned by only 10% of treated patients and one patient noted, “I use the word neuropathy with people who know what it means” (Participant 8109).

Crisis pain occurred in 60% of treated patients and 29.4% of treatment-naïve patients and were described as recurring, sudden attacks of pain of varying duration. Pain would move up the body for one patient whose crisis pain began in the hands and feet and noted that “every fifth burning dart or so, it goes up to my elbows and up to my knees” (Participant 8103). Patients also described pain in specific locations, primarily in the extremities. For one patient, pain occurred intermittently and was described as “somebody is just stabbing me with a sharp object, you know just consistently. And that pain will last approximately I will say ten or fifteen, maybe thirty seconds. It would go away, then it would come back” (Participant 6102). Patients also reported tingling, described as “somebody lit a sparkler in my feet and my hands” (Participant 8001). A patient noted that the tingling varied in severity, stating “mildly, it's a feeling of when you know your hand falls asleep. And when it's actually painful … it's like a burning” (Participant 7002).

##### Temperature intolerance

85% of treated patients and 47.1% treatment-naïve patients reported experiencing heat intolerance, the most frequently experienced concept within this domain. Heat intolerance was experienced typically at 90 degrees Fahrenheit or higher, and was described by a patient as a tingling feeling on the skin which caused the patient to feel “sluggish … I feel like I'm coming down with the flu” (Participant 7001). Many patients would cope with consistent use of air conditioning and avoiding going outdoors on hot days. Additionally, some patients reported experiencing both heat and cold intolerance. One patient would “turn my heater on at work even in the summer because I’m always freezing” (Participant 8102). Lastly, 65% of treated patients and 52.9% of treatment-naïve patients reported a lack of sweating. A patient noted the differences in amount of sweat compared to others, stating “if their armpits might be soaking wet and their chest might be wet and it might be visible through their shirt … hardly any sweat there on my skin” (Participant 8106).

##### Low energy

Many patients reported feeling tired (90% treated, 76.5% treatment-naïve) and fatigued (70% treated, 47.1% treatment-naïve). One patient who experienced both symptoms described fatigue as “my body just feels like it doesn't have the energy to continue doing that activity and my arms and legs feel heavy” whereas tiredness was associated with the need to sleep, such as “if we get in the car, I'm usually sleeping within five minutes. In class, I would always just put my head down and fall right asleep” (Participant 3103). Another patient described fatigue as running out of energy for daily activities and “get to where I start taking short cuts” (Participant 6104). While running errands, this patient would “sit in the truck for thirty minutes just to get up enough energy to go in and get the groceries” (Participant 6104). For many patients, taking breaks and naps throughout the day were necessary to have enough energy to get through the day.

##### Digestive/gastrointestinal symptoms

Eighty percent of treated patients and 58.8% of treatment-naïve patients experienced GI symptoms, including diarrhea, constipation, and stomach pain. A patient noted that his/her stomach pain was particularly severe, stating that “I noticed that I would get like trapped gas to the point to where I would end up in the emergency room for my stomach because it wouldn't go anywhere, it would just sit there and then the ER gave me every medicine possible and as much as they could give me to try and relieve the pain, but it did nothing” (Participant 3002). Another patient experienced alternating constipation and diarrhea, noting that “constipation will usually last a couple days… And then that turns into diarrhea. And then I feel like I just flush out my system in one day” (Participant 3103). For some patients, their GI symptoms required significant planning or avoidance of daily activities. One patient noted that he/she would not plan long activities, stating “I can't do it … I wouldn't want to do it if I had diarrhea or something like that” (Participant 3101). Another patient with severe GI symptoms stated, “I would have gas, unbelievable gas that I could not get rid of. … I just had so much pain. I couldn't go anywhere” (Participant 2101). This patient expressed concern that the consistent constipation and diarrhea was so severe that “there is something else got to be going wrong. … it's dysfunctional” (Participant 2101).

##### Hearing and vision impairment

A number of patients experienced some to total hearing loss (75% treated, 17.6% treatment-naïve), with or without tinnitus. A patient who experienced both hearing loss and tinnitus stated “I lost like fifty percent of my hearing. And the ringing in my ears, most of the time when I wake up in the morning, the ringing is like, is really like twice as loud as it normally is” (Participant 6104). The patient sat in a car in the driveway during the interview, stating “I didn't want any distractions. Because if any, if I hear, if there is any noise in the room anywhere, then I can't hear you” (Participant 6104). Fifty percent of treated patients and 17.6% of treatment-naïve patients experienced vision impairment which similarly varied in severity. One patient described seeing halos and glowing lights, which caused discomfort and watery eyes. Another patient occasionally experienced double vision, stating “If I close one eye I’m fine but with the two I walk like I’m drunk …it just starts out like almost instantaneously I’m seeing double” (Participant 3104). Lastly, a patient who had greater sight impairment lost vision in one eye for 10 days, describing swelling and pain whenever he/she moved the eye. Half of the vision was lost, described as “if you were looking at TV it would, half of it would go gray” (Participant 3101).

##### Most severe and bothersome symptoms

The most severe symptoms as reported by the patients (N = 37) (rated from 0 = none to 10 = extremely severe) were neuropathic pain (mean score 8.3, n = 9), stomach pain (mean score 8.0, n = 5), burning pain (mean score 7.7, n = 15), and fatigue (mean score 7.2, n = 13).

The most bothersome symptoms (rated from 0 = not bothersome at all to 10 = extremely bothersome) were stomach pain (mean score 9.8; n = 5), breathing difficulty (mean score 9.3, n = 4), fatigue (mean score 8.2, n = 14), neuropathic pain (mean score 7.9, n = 6), and constipation (mean score 7.9, n = 6). Table 3 includes the severity and bother ratings for all symptoms in treated and treatment-naïve patients. While neuropathic pain and burning pain were described in conceptually similar terms, these symptom ratings are presented separately to reflect the terminology used by patients.

#### Impacts of FD

Nearly all of the patients who were interviewed expressed impacts related to FD. Ninety-five percent of treated patients reported experiencing at least one impact in each of the following domains: physical activity limitations, difficulty with daily activities, social and lifestyle limitations, and emotional health. When asked to describe how FD impacted their daily lives, the most frequent impacts reported were in the work/school limitations domain for both treated (85%) and treatment-naïve (65%) patients. Other predominant impact domains were exercise limitations (80%), social activity (75%), and difficulty with household responsibilities (75%) for treated patients. For treatment-naïve patients, the most frequently reported impacts were depression (59%), memory problems (59%), worry/fear (54%), and exercise limitations (54%). Notably, a number of patients in both groups expressed significant emotional impacts, including stress, concerns about perception of health, and difficulty with self-image.

Patients were asked to rate how difficult each impact was to cope with (ranging from 0 = not difficult at all to 10 = extremely difficult). The impacts with the highest difficulty ratings were stress, irritability, limited outdoor activity, and guilt. Table 4 presents the impact frequency and difficulties ratings for treated and treatment-naïve patients.

**Table 4:** Impact frequencies and difficulty ratings

Impact concepts	Impact frequency, n (%)			Impact difficulty rating, mean (n)
	Treated group (n = 20)	Treatment-naïve group (n = 17)	All (N = 37)	All (N = 37)
*Physical activity limitations and restrictions*				
General physical limitations	*9 (45.0)*	4 (23.5)	*13 (35.1)*	*7.0 (6)*
Exercise limitations	**16 (80.0)**	*9 (52.9)*	*25 (68.6)*	*5.0 (21)*
Difficulty with stairs	*9 (45.0)*	3 (17.6)	12 (32.4)	*6.2 (6)*
Walking limitations	*7 (35.0)*	*7 (41.2)*	*14 (37.8)*	*6.9 (8)*
*Difficulty doing daily activities*				
General daily activity limitations	*11 (55.0)*	*8 (47.1)*	*19 (51.4)*	*6.0 (4)*
Difficulty with driving	3 (15.0)	0	0	–
Difficulty with household responsibilities	**15 (75.0)**	6 (35.3)	*21 (56.8)*	*6.2 (18)*
Difficulty with personal care	7 (35.0)	1 (5.9)	8 (21.6)	**7.0 (4)**
Work/school limitations	**17 (85.0)**	*11 (64.7)*	**28 (75.7)**	*6.3 (24)*
*Social/lifestyle limitations and restrictions*				
Clothing choices	*8 (40.0)*	3 (17.6)	11 (29.7)	*5.0 (3)*
Diet restrictions	7 (35.0)	5 (29.4)	12 (32.4)	*6.8 (4)*
Leisure	*9 (45.0)*	5 (29.4)	*14 (37.8)*	**7.3 (8)**
Relationships				*6.9 (16)*
Family relationships	*11 (55.0)*	5 (29.4)	0	–
Friend relationships	3 (15.0)	4 (23.5)	0	–
General relationship	3 (15.0)	1 (5.9)	0	–
Spouse relationship	5 (25.0)	4 (23.5)	0	–
Outdoor activity	*7 (35.0)*	2 (11.8)	9 (24.3)	**9.5 (2)**
Reproductive choices	4 (20.0)	0	4 (10.8)	**9.0 (2)**
Sexual activity	3 (15.0)	4 (23.5)	7 (18.9)	**7.9 (7)**
Social activity	**15 (75.0)**	*7 (41.2)*	*22 (59.5)*	**6.5 (15)**
*Emotional health*				
Anger	0	2 (11.8)	2 (5.4)	*6.0 (1)*
Depression	*10 (50.0)*	*10 (58.8)*	*20 (54.1)*	*7.1 (14)*
Embarrassment	5 (25.0)	3 (17.6)	8 (21.6)	*4.4 (5)*
Frustration	*8 (40.0)*	4 (23.5)	12 (32.4)	**7.8 (4)**
Guilt	4 (20.0)	2 (11.8)	6 (16.2)	**9.0 (2)**
Perceptions of health	*8 (40.0)*	*6 (35.3)*	*14 (37.4)*	*7.7 (10)*
Irritability	*7 (35.0)*	1 (5.9)	8 (21.6)	**10.0 (1)**
Negative outlook	3 (15.0)	1 (5.9)	4 (10.8)	3.3 (3)
Positive outlook	5 (25.0)	0	0	–
Self-image	*11 (55.0)*	2 (11.8)	13 (35.1)	2.0 (1)
Stress	*10 (50.0)*	4 (23.5)	*14 (37.8)*	**10.0 (2)**
Worry/fear	*10 (50.0)*	*9 (52.9)*	*19 (51.4)*	6.6 (7)
*Sleep difficulties*				
Difficulty falling asleep	*7 (35.0)*	3 (17.6)	10 (27.0)	*7.0 (2)*
Difficulty staying asleep	6 (30.0)	5 (29.4)	11 (29.7)	*7.5 (2)*
Sleep quality	5 (25.0)	*8 (47.1)*	*13 (35.1)*	*6.6 (10)*
*Cognitive function*				
Memory problems	*13 (65.0)*	*10 (58.8)*	*23 (62.2)*	*6.2 (12)*
Poor focus/concentration	*10 (50.0)*	*8 (47.1)*	*18 (48.6)*	**7.7 (15)**
Other cognitive limitations	6 (30.0)	0	0	–
*Other impacts*				
Other	3 (15.0)	0	3 (8.1)	**7.0 (5)**

Italics indicate higher impact frequency and difficulty. Bold indicate highest impact frequency and difficulty

Difficulty ratings range from 0 = not difficult at all, to 10 = extremely difficult

##### Work and school

Many patients noted that their FD symptoms, including pain crises, tinnitus, and fatigue, impacted their ability to work or be productive at work and/or school. One patient “lost two jobs because I was fatigued, and I would get pain crisis and lay on the floor in the break room” (Participant 3103). Another patient who experienced sudden pain crises at work noted that “it just comes on really fast where I feel sick and I run a fever and then I'm like in crippling pain … I can't even walk out of work, which is embarrassing, so I have to be carried because my feet hurt so bad” (Participant 8109). Another patient’s tinnitus affected his/her ability to listen to others at work, stating “I have to be very alert and attentive to [redacted] and it’s very hard to hear what they’re saying to me when the ringing starts” (Participant 2002).

##### Physical activities

Heat intolerance was one of the key symptoms that affected patients’ physical activities. While roller skating, one patient mentioned that “I always had to immediately take my skates off … It could be the dead of winter and I would walk on the cement to cool my feet off. It felt like they were on fire” (Participant 2101). Similarly, another patient mentioned participating less in physical activities because “I feel like I’m overheating … I need to manually cool myself down quite often” (Participant 8103). Other symptoms that impacted physical activities included fatigue, neuropathic pain, and burning pain. A patient noted that he/she would feel more fatigued than usual during physical activities, describing it as “my legs and my whole body just feels like feel like I’m about 90 years old” (Participant 8110).

##### Social activities

Patients reported a number of limitations in the types of social activities that they were able to participate in as well as relationships with others. For one patient, the extent of his/her social activities depended on whether others knew about the patient’s condition, stating “I won't go running or work out with people … I don't wanna sit and explain it” (Participant 7002). Many linked the impact on social activities to symptoms of fatigue and pain. A patient noted that it was difficult for friends to understand that he/she didn’t have the same amount of energy as others might, and so they would “wonder if I’m mad at them, or am I mad at the world … It is hard not to be isolated or isolate yourself” (Participant 8109). Some patients chose to limit themselves to close relationships, such as one patient who stated “I don’t even try to have a relationship like a friendship or relationship with anyone other than my family … it’s been bothersome with the pain and the ringing in the ear and the burning because I can’t do what the average person could do” (Participant 2002).

### FD-PRO development

Based on the data acquired through the targeted literature search, the KOL interviews, and the CE interviews, an initial draft of the FD-PRO was developed. The initial draft contained the following concepts: pain, burning, numbness or tingling (assessed separately for hands and feet), abdominal pain, abdominal discomfort, headache, cold and heat intolerance, swelling in the lower extremities, tinnitus, tiredness/fatigue, hearing impairment, exposure to activities that would lead to sweating, and lack of sweating.

### Cognitive debriefing interviews

The draft FD-PRO was evaluated through 3 waves of cognitive interviews with a total of 14 patients, with changes made to the draft FD-PRO after each wave. Of the 14 patients, 11 had received treatment for ≥ 6 months, 1 had received treatment for ≤ 6 months, and 2 were treatment-naïve. As shown in Table 2, the average age of patients was 48.9 years, 53% were female, and all were White.

Changes made based on the first wave of interviews (n = 4) included improving the specificity of the intended recall period to “the past 24 h”; changing “burning” to “burning feeling”; and adding descriptors for the location of abdominal pain/discomfort. Changes made based on the second wave of interviews (n = 5) included adding “arms” with “hands” and “legs” with “feet” on several items to include the entire extremity; adding an item on vision impairment; and revising the descriptors of the location of abdominal pain/discomfort to provide alternatives for wave 3 testing. A final 5 patients evaluated the FD-PRO in wave 3, during which the revised terminology was confirmed. Patients indicated no difficulty in comprehension of the items and confirmed their relevance, and one of the alternative descriptors for the location of abdominal pain/discomfort was chosen. After the final wave of interviews, the selected items were pain, burning, numbness or tingling (assessed separately for hands or arms and feet or legs), abdominal pain, headache pain, heat intolerance, swelling in the feet or legs, tinnitus, tiredness/fatigue, hearing impairment, visual impairment, exposure to activities that would lead to sweating, lack of sweating, and engaging in physical activities.

## Discussion

In FD, the progressive accumulation of GL-3 and other products in lysosomes causes a constellation of signs and symptoms that can start as early as childhood and gradually progress to organ failure and death [1, 2]. Early signs and symptoms include neuropathic and GI pain, corneal verticillata, angiokeratoma, temperature intolerance, fatigue, and audiovisual issues [1, 2]. However, there has been no systematic way to evaluate the frequency and impact of these signs and symptoms, or to identify which are the most debilitating and most important in terms of quality of life. Thoroughly understanding patient experiences is key in measuring the burden of FD. This study was designed to comprehensively evaluate the symptoms and functional limitations experienced by FD patients and to develop a PRO measure that can be used to assess patient experiences.

This study was done using robust methodology in accordance with the FDA guidance and expert guidelines for establishing content validity [3, 8]. The conceptual framework was developed based on a targeted literature review, the advice of KOLs, and input from patients regarding the signs and symptoms that they most frequently experience and the impact on daily lives. Both treated and treatment-naïve patients and both male and female patients were included in the CE interviews, and the results demonstrated that these patient subgroups had broadly similar experiences, although treatment-naïve patients (who were generally in an earlier stage of disease progression) had fewer and milder signs and symptoms. The concept items were then evaluated and adjusted through cognitive interviews with treated and treatment-naïve patients. The interviews assessed patients’ understanding of the concepts and resulted in the development of a disease-specific instrument that targets the signs and symptoms of FD and their impact on patients.

The symptoms most commonly reported by patients, regardless of sex or treatment status, were neuropathic symptoms, temperature intolerance, energy difficulties, digestive/GI symptoms, and hearing/vision impairment. The most severe symptoms reported by patients were neuropathic pain, stomach pain, burning pain, and fatigue, and the most bothersome symptoms were stomach pain, breathing difficulty, fatigue, neuropathic pain, and constipation. Diarrhea and constipation were not included in the FD-PRO because assessment of these experiences may need to occur episodically (e.g., after every bowel movement), which is not possible with the daily FD-PRO. Additionally, standard measures, such as the Bristol Stool Form Scale, exist to measure these GI symptoms [11]. Clinicians and researchers should consider these supplemental measures of diarrhea and constipation when administering the FD-PRO.

When asked how FD impacted their daily lives, patients reported that work/school limitations were the most common impacts, regardless of whether they were treated or treatment-naïve. Other common impacts were exercise limitations, social activity, and difficultly with household responsibilities in treated patients and depression, memory problems, worry/fear, and exercise limitations in treatment-naïve patients. The impacts with the highest difficulty ratings were stress, limited outdoor activity, and guilt.

The strengths of this study lie in the robust methodology used to develop the FD-PRO and incorporating the patient perspective of the constellation of symptoms that characterize FD. The spectrum of symptoms assessed by the FD-PRO is especially important in accounting for the heterogeneity of disease presentation, as it is well documented that symptoms vary by patients and phenotype. The FD-PRO is complementary to other PROs that assess specific symptoms of FD, such as the Brief Pain Inventory to assess neuropathic pain or the Bristol Stool Form Scale to assess GI symptoms. At the time of this publication, other PROs have been developed that assess specific components of FD. These include the FABry Disease Patient-Reported Outcome-GastroIntestinal which evaluates FD-related GI signs and symptoms (abdominal cramps, bloating, and diarrhea); the Fabry-Specific Pediatric Health and Pain Questionnaire which evaluates the frequency of disease-specific symptoms in children; and the Patient Needs Questionnaire Fabry which assesses patient needs and expectations towards their treatment [5–7]. These FD questionnaires are narrow in scope or population, which impact the ability to capture the broader spectrum of symptoms of FD. In contrast, the FD-PRO assesses a range of FD symptoms, making it appropriate for use in clinical trials, medical practice, or for patients to monitor their symptoms and disease progression.

Limitations include the potential that some signs and symptoms reported by patients in the treated group might instead be side effects of ERT. Indeed, some symptoms were reported more commonly in treated patients, and these were retained for quantitative testing. It was also hypothesized that the symptom pattern would differ between male and female patients. However, while there were numerical differences between male and female patients for several symptoms, the overall pattern was very similar between the sexes. Patient recall can be a limitation in any patient-reported instrument, so questions on the FD-PRO were phrased to focus on the “past 24 h” to address this potential limitation. Finally, the number of items in the FD-PRO may pose a burden that would influence the accuracy or completeness of patient reports. It is anticipated that the results of quantitative testing will be key in adjusting the final number of items in the FD-PRO.

The next step in the development of the FD-PRO will be to evaluate its psychometric properties in terms of reliability and validity, and to assess patient compliance and burden in using the instrument.

## Data Availability

Data generated from this study are available from the corresponding author and with permission of Sanofi Genzyme on reasonable request.
